# The potential clinical value of contrast-enhanced echocardiography beyond current recommendations

**DOI:** 10.1186/s12947-015-0045-0

**Published:** 2016-01-05

**Authors:** Malin K. Larsson, Cristina Da Silva, Elif Gunyeli, Ali Akebat Bin Ilami, Karolina Szummer, Reidar Winter, Anna Bjällmark

**Affiliations:** 1 Department of Medical Engineering, School of Technology and Health, KTH Royal Institute of Technology, Alfred Nobels Allé 10, SE-141 52 Huddinge, Sweden; 2 Department of Cardiology, Karolinska University Hospital, Hälsovägen B52-54, SE-141 86 Stockholm, Sweden; 3 Department of Molecular Medicine and Surgery, Karolinska Institutet, K1 Klinisk fysiologi, N2:01, SE-171 76 Stockholm, Sweden

**Keywords:** Contrast agent, Echocardiography, Wall motion score index, Ejection fraction

## Abstract

**Background:**

Contrast agents are used in resting echocardiography to opacify the left ventricular (LV) cavity and to improve LV endocardial border delineation in patients with suboptimal image quality. If a wider use of contrast-enhanced echocardiography would be adopted instead of the current selective approach, diagnoses such as myocardial ischemia and LV structural abnormalities could potentially be detected earlier. The aim was therefore to retrospectively investigate if contrast-enhanced echocardiography beyond the current recommendations for contrast agent usage affects assessment of wall motion abnormalities, ejection fraction (EF) and detection of LV structural abnormalities. A secondary aim was to evaluate the user dependency during image analysis.

**Methods:**

Experienced readers (*n* = 4) evaluated wall motion score index (WMSI) and measured EF on greyscale and contrast-enhanced images from 192 patients without indications for contrast-enhanced echocardiography. Additionally, screening for LV structural abnormalities was performed. Repeated measurements were performed in 20 patients by the experienced as well as by inexperienced (*n* = 2) readers.

**Results:**

Contrast analysis resulted in significantly higher WMSI compared to greyscale analysis (*p* < 0.003). Of the 83 patients, classified as healthy by greyscale analysis, 55 % were re-classified with motion abnormalities by contrast analysis. No significant difference in EF classification (≥55 %, 45–54 %, 30–44 %, < 30 %) was observed. LV structural abnormalities, such as increased trabeculation (*n* = 21), apical aneurysm (*n* = 4), hypertrophy (*n* = 1) and thrombus (*n* = 1) were detected during contrast analysis. Intra- and interobserver variability for experienced readers as well as the variability between inexperienced and experienced readers decreased for WMSI and EF after contrast analysis.

**Conclusions:**

Contrast-enhanced echocardiography beyond current recommendations for contrast agent usage increased the number of detected wall motion and LV structural abnormalities. Moreover, contrast-enhanced echocardiography increased reproducibility for assessment of WMSI and EF.

## Background

Accurate assessment and high reproducibility of left ventricular (LV) volumes, ejection fraction (EF), and myocardial wall motion are important factors for diagnosis of cardiac diseases [1, 2]. Cardiac ultrasound (echocardiography) is currently the most widely used imaging modality in cardiac diagnostic imaging due to high cost effectiveness, wide availability in hospitals, generation of real-time images, non-ionization radiation and high feasibility. On the other hand, the image quality is suboptimal in some patients, which leads to relatively low accuracy and reproducibility compared to analysis based on magnetic resonance images in these cases [3]. To overcome this problem, it has been demonstrated that the introduction of a contrast agent during echocardiography improves endocardial border delineation, and hence improves the accuracy of LV volume and EF measurements as well as myocardial wall motion score index (WMSI) evaluations [4, 5]. Moreover, contrast-enhanced echocardiography has shown to reduce inter- and intraobserver variability [4, 6].

Current guidelines from both the European and American Societies (European Association of Cardiovascular imaging (EACVI) and American Society of Echocardiography (ASE)) recommend the use of contrast-enhanced echocardiography in patients with suboptimal image quality or when there is a suspicion of structural abnormalities [7, 8]. In spite of these recommendations, there appears to be an underuse of contrast agents during echocardiographic examinations. It has been proposed that approximately 10 to 15 % of all cardiac patients have indications for contrast-enhanced echocardiography [5, 6]. Nevertheless, a recent study [9] demonstrated that only 3.8 % of the echocardiographic examinations included a contrast agent during image acquisition. The underuse of contrast agent in clinical practice may alter the diagnosis and treatment strategies for the patients, as diagnostic indications can be omitted. It can be hypothesized that if a wider use of contrast-enhanced echocardiography would be adopted instead of the current selective approach, diagnoses such as myocardial ischemia and LV structural abnormalities may be detected earlier. Moreover, routine use of a contrast agent during echocardiography in all patients might also reduce the user dependency during image analysis, as the image quality increases, not only in patients with suboptimal image quality, but also in patients with good image quality without current indications for contrast-enhancement. The aim of this study was therefore to retrospectively investigate if contrast agent usage during echocardiography beyond the current recommendations affects the evaluation of cardiovascular function with respect to: 1) Assessment of regional wall motion abnormalities, 2) EF classification, and 3) Detection of LV structural abnormalities. A secondary aim was to evaluate if contrast-enhanced echocardiography reduced the inter- and intraobserver variability on LV volume and EF measurements as well as WMSI evaluations, irrespective of the reader’s experience level of contrast-enhanced echocardiography.

## Methods

### Eligible patients

This retrospective study included patients who were referred to stress echocardiography from January 2013 to February 2014 at the Karolinska University Hospital in Huddinge (The heart clinic), Sweden. This patient group was selected as the image protocol at our institution routinely involves analysis of both greyscale and contrast-enhanced images both at rest and during stress, regardless of image quality. This enables a comparison of the diagnostic outcome between greyscale and contrast-enhanced images *at rest* in the subset of patients beyond the current recommendations for contrast agent usage in standard echocardiography. Note that the greyscale images routinely are acquired with such quality that deformation analysis using speckle tracking is feasible during rest and stress. The image acquisition was performed with a GE Vivid E9 (GE Healthcare, Wisconsin, USA), at a transmitted frequency of 1.5–1.7 MHz. All contrast-enhanced images were acquired at a low MI index using contrast specific image sequences, such as pulse inversion. SonoVue® (Bracco Diagnostics Inc., Switzerland) (*n* = 187) and Optison® (GE Healthcare, Wisconsin, USA) (*n* = 5) were used as contrast agents.

The exclusion criteria were based on the recommendations for contrast agent usage during standard echocardiography [7, 8]. Patients were excluded if they fulfilled the criteria for contrast-enhanced echocardiography, i.e. two or more contiguous LV segments not visualized on a greyscale image or suspicion of LV structural abnormalities, such as apical hypertrophic cardiomyopathy, ventricular non-compaction, thrombus or ventricular pseudoaneurysm. Experienced echocardiographer (*n* = 4, 3–15 years of experience) of contrast-enhanced echocardiography visually evaluated whether patients should be included in the study or not. Additionally, patients were excluded if any apical 2 chamber (ch), 3ch or 4ch images in either greyscale or contrast mode were missing. The study protocol was approved by the regional ethical review board in Stockholm, Sweden.

### Image analysis

**Fig. 1:**
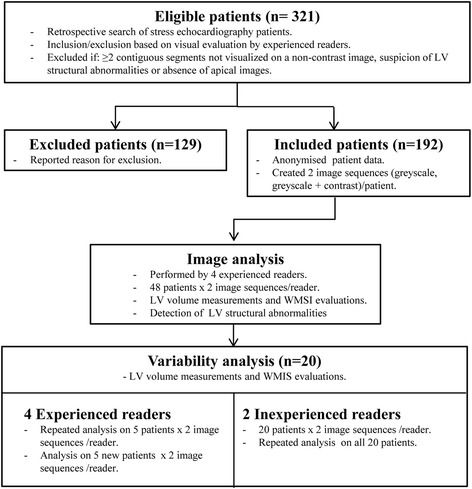
Schematic illustration of the image analysis process

The flow chart of the image analysis is shown in Fig. 1. For all patients included, two image sequences (greyscale images, greyscale + contrast images) were prepared for each patient. The latter image sequence contained both modes to support the evaluation of LV structural abnormalities, but only the contrast images in this sequence were used during WMSI and EF assessment. Before image analysis was initiated, all patient data were anonymised. The image analyses were allocated between four experienced readers and the readers evaluated the two image sequences of one patient with a minimum of two days apart (greyscale images, greyscale + contrast images). Furthermore, repeated analyses were performed on five patients for each of the experienced readers, who were blinded to previous results. To assess the interobserver variability, these five patients were also analyzed once by another experienced reader. Moreover, the variability between readers with different experience level was evaluated by letting two inexperienced readers do repeated analyses on the same twenty patients as the ones being re-evaluated by the experienced readers. The inexperienced reviewers had less than 0.5 years of experience of analyzing echocardiographic images. Prior to initiation, the inexperienced readers received a demonstration of the image analysis process.

#### Assessment of wall motion

From apical 2ch, 3ch and 4ch views, WMSI was obtained by dividing the LV using an 18 segment model [10]. Each segment was assigned a wall motion score (WMS) depending on the myocardial thickening and movement pattern. A normally contracting segment was graded as 1, hypokinesia as 2, akinesia as 3 and dyskinesia as 4. A segment was given NA if evaluation of that specific segment was not applicable due to insufficient image quality.

#### LV volume measurements

Left ventricular end-diastolic volume (EDV), left ventricular end-systolic volume (ESV) and EF were measured in one cardiac cycle using the biplane method of disks summation (the modified Simpson’s rule) in EchoPAC (GE Healthcare, Wauwatosa, WI, USA). End-diastole was visually defined as the maximal cavity area following mitral valve closing, whereas end-systole was defined as the minimal cavity area preceding mitral valve opening. In accordance with the recommendations from EACVI and ASE, papillary muscle and trabeculations were excluded from the cavity during tracing [10].

#### Detection of LV structural abnormalities

The contrast-enhanced images (3 images/patient) were screened for LV structural abnormalities such as apical hypertrophic cardiomyopathy, ventricular non-compaction, thrombus and ventricular pseudoaneurysm. The screening for LV structural abnormalities was only conducted by the experienced readers. Note that patients, who showed structural abnormalities on the greyscale images, were already excluded from the study since they had indication for contrast-enhanced echocardiography.

### Statistics

Data analysis was performed using the statistical software IBM SPSS Statistics 19 (Armonk, New York, USA). Age, blood pressure, LV volumes and EF were classified as continuous data and expressed with mean and standard deviation. Paired Student *t* test (95 % confidence level) was used to compare numerical groups. WMSI for each patient was determined by calculating the sum of all WMS divided by the number of visualized segments [10]. Paired Student *t* test (95 % confidence level) was used to compare WMSI before and after contrast. Patients were categorized according to reference values of EF: ≥ 55 % reference range, 45–54 % mildly abnormal, 30–44 % moderately abnormal, < 30 % severely abnormal [10]. Additionally, the difference in EF classification between greyscale and contrast image analysis was investigated using McNemar test. Intra– and interobserver variability for WMSI and EF measurements was assessed by using intra-class correlation coefficient (ICC) (95 % confidence level).

## Results

### Eligible patients

In total, 321 patients referred for stress echocardiography at the Karolinska University Hospital in Huddinge, Sweden, were screened for the study. After the review process, 192 patients were included in the patient group, whereas 129 patients were excluded due to insufficient image quality (80), allergy to contrast agent (1), absence of at least one apical image view (30), suspicions of non-compaction (10), thrombus (7) or hypertrophic cardiomyopathy (1). Patient characteristics of the patient group included in the study are presented in Table 1.

**Table 1:** Patient characteristics

Age (years)	65 ± 13	
Sex (female/male)	62/130	
BMI (kg/m^2^)	27 ± 4^a^	
Systolic blood pressure (mmHg)	133 ± 23^b^	
Diastolic blood pressure (mmHg)	73 ± 11^b^	
Indication for stress echocardiography		
Ischemic heart disease		171 (89 %)
Low-flow/low-gradient aortic stenosis		5 (3 %)
Ischemic heart disease and low-flow/low-gradient aortic stenosis		6 (3 %)
Follow-up heart transplantation		9 (5 %)
Arrhythmia		1 (<1 %)

Patient characteristics of the patient group included in the study (*n* = 192). Continuous variables are expressed as mean ± standard deviation

^a^ = mean from 182 patients^b^ = mean from 137 patients

### Image analysis

#### Assessment of wall motion

Table 2 shows a comparison of all segments’ WMS for greyscale and contrast images. As can be seen, the ability to perform quantitative analysis was considerably improved with contrast, as the number of unanalyzed segments reduced from 169 (4.9 %) to 14 (0.4 %) segments when evaluating contrast images instead of greyscale images. Compared to greyscale analysis, 432 segments were classified with higher WMS (NA segments not included) after contrast analysis. Of these segments, the most frequently observed change in WMS was a shift from normal to hypokinesia. Furthermore, 255 segments were classified with lower WMS after contrast analysis compared with greyscale analysis.

**Table 2:** Wall motion score

	WMS – Contrast				
WMS - Greyscale	NA	1	2	3	4
NA	6	120	35	6	2
1	6	2311	345	27	0
2	2	185	207	44	6
3	0	27	38	73	10
4	0	1	2	2	1

Distribution of the myocardial segment wall motion score (WMS) (1-4 or NA) obtained for greyscale and contrast-enhanced image analysis by experienced readers

*NA* not applicable, *1* normal, *2* hypokinesia, *3* akinesia, *4* dyskinesia

**Fig. 2:**
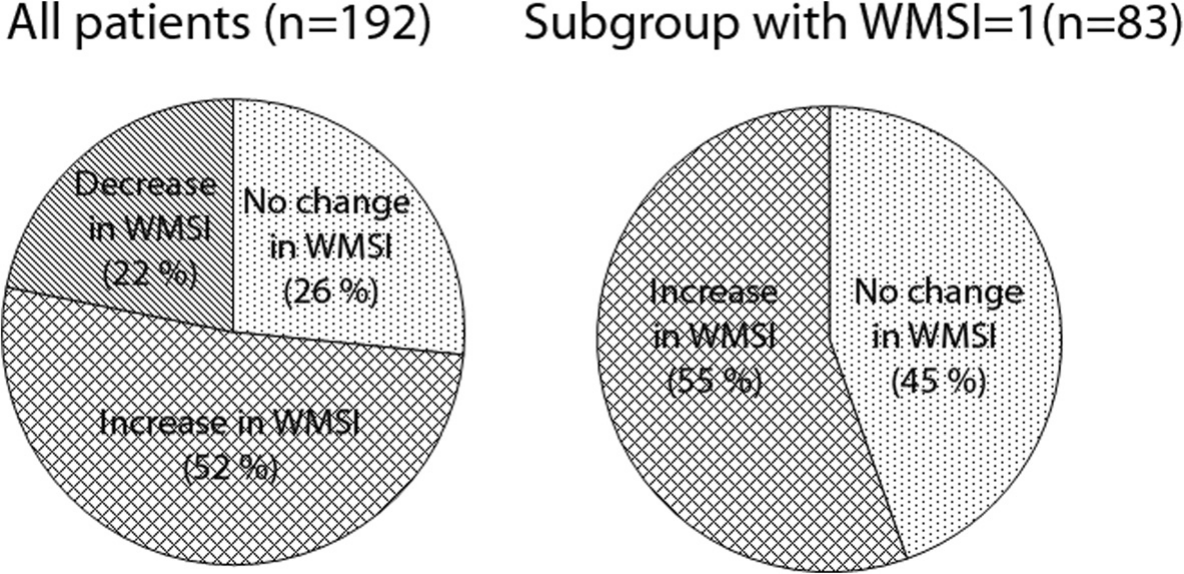
The change in wall motion score index (WMSI) when comparing contrast analysis with greyscale analysis for the whole patient group (left) and the subgroup without wall motion abnormalities (right)

When investigating WMSI at patient level, the WMSI obtained after analysis of contrast-enhanced images was significantly higher compared to greyscale image analysis (WMSI = 1.23 vs. WMSI = 1.29 *p* < 0.003). This corresponds to 99 patients having increased WMSI, 42 patients having decreased WMSI and 51 patients with no change after contrast analysis (Fig. 2). For those patients with increased WMSI after contrast analysis, higher WMS was observed in 23 % (SD 15 %) of the segments that were visualized during greyscale analysis. Additionally, for those patients with decreased WMSI after contrast analysis, lower WMS was observed in 29 % (SD 15 %) of the visualized segments during greyscale analysis.

Of the 192 patients included in the study, 83 patients (43 %) were classified with normal wall motion after image analysis on greyscale images, i.e. WMS = 1 for every segment. Patients with NA segments were not included in these 83 patients, implying that, based on greyscale analysis, these patients were observed without wall motion abnormalities. After image analysis of contrast-enhanced images, 46 (55 %) of these patients were reclassified with abnormal wall motion (Fig. 2). 125 segments with abnormal wall motion were identified in the left anterior descending (LAD) regions, 25 segments were identified in the right coronary artery (RCA) regions and 51 segments were identified in the circumflex (CX) coronary artery regions [10]. Segments with variable perfusion (RCA/CX, LAD/CX and RCA/LAD) were counted twice. In average, 18 % (SD 10 %) of the segments in the LV received increased WMS after contrast analysis in these patients.

#### LV volume measurements

There was a significant difference in LV volumes between greyscale and contrast-enhanced image analysis. Both EDV and ESV were increased during presence of contrast agent, see Table 3.

**Table 3:** Volume measurements

EDV greyscale vs EDV contrast (ml)	98 ± 38 vs 119 ± 44*
ESV greyscale vs ESV contrast (ml)	46 ± 29 vs 53 ± 37*
EF greyscale vs EF contrast (%)	56 ± 12 vs 59 ± 14*

Mean left ventricular volumes and EF obtained after image analysis (*n* = 192). Significant difference (*p* < 0.05) between greyscale and contrast-enhanced images is marked with*

*EDV* end-diastolic volume, *ESV* end-systolic volume, *EF* ejection fraction

**Fig. 3:**
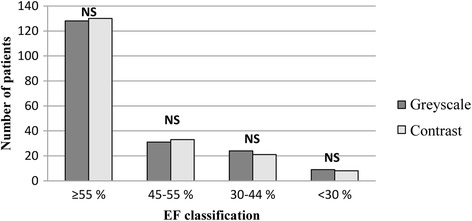
Distribution of patients according to their ejection fraction (EF). Dark grey bars represent the distribution for greyscale images whereas light grey bars represent the distribution for contrast-enhanced images. NS indicates a non-significant distribution between the different imaging modes

Table 4 shows a comparison of the patients’ EF classification for greyscale and contrast-enhanced images. As can be seen, a majority of the patients (139 patients (72 %)) remained in the same diagnostic range for both imaging modes. For the rest of the patients, there was a trend towards higher EF (30 patients (15 %) compared with 23 patients (12 %)) after contrast analysis. As Fig. 3 shows, there was no significant difference in EF classification between the two imaging modes.

**Table 4:** Ejection fraction

	EF – Contrast			
EF - Greyscale	≥55 %	45–54 %	30–44 %	<30 %
≥55 %	112	12	4	0
45–54 %	14	13	4	0
30–44 %	4	8	9	3
<30 %	0	0	4	5

Distribution of the measured ejection fraction (EF) for each patient for greyscale and contrast-enhanced image analysis by experienced readers

*EF* ejection fraction

#### Detection of LV structural abnormalities

The contrast-enhanced images revealed increased trabecula formations within the LV in 21 patients, indicating an enhanced risk of non-compaction. Moreover, apical aneurysms were detected in four patients, while hypertrophy and apical thrombus were detected in one patient each.

#### Variability analysis

Contrast-enhancement image analysis improved the agreement for all three conditions of variability for both WMSI and EF analysis, see Table 5. The improvement was most pronounced for WMSI evaluations performed by different experienced readers, with a change from 0.61 to 0.87 after contrast agent injection.

**Table 5:** Variability analysis

	Experienced readers analysis 1 vs analysis 2	Experienced readers reader 1 vs reader 2	Experienced readers vs inexperienced readers
WMSI	(*n* = 20)	(*n* = 20)	(*n* = 40)
Greyscale	0.89	0.61	0.58
Contrast	0.94	0.87	0.81
EF	(*n* = 20)	(*n* = 20)	(*n* = 40)
Greyscale	0.90	0.80	0.77
Contrast	0.98	0.95	0.94

Intra-class correlation coefficient for wall motion score index (WMSI) and ejection fraction (EF)

## Discussion

The main finding from this study was that contrast-enhanced echocardiography significantly altered the assessment of cardiac function compared with greyscale echocardiography, in a patient group specifically selected due to not having an indication for contrast enhancement. This was mainly driven by an improved detection of regional wall motion abnormalities, evidenced by the fact that WMSI was significantly higher after contrast analysis compared with greyscale analysis. This was further evident when only considering patients (*n* = 83) with normal wall motion (WMS = 1) and sufficient greyscale image quality (no NA segments) in every segment, where as much as 55 % of these patients, previously classified without wall motion abnormalities, were re-classified having regional wall motion abnormalities after contrast-enhanced analysis. Keeping in mind that the addition of contrast agent also allowed for detection of LV structural abnormalities in 27 patients, emphasizes that a broader use of contrast-enhanced echocardiography can contribute to earlier detection of cardiovascular diseases.

Compared to these previous studies, the present study strictly selected patients without indications for contrast agent usage during standard echocardiography. Consequently, the clinical effect of contrast-enhanced echocardiography could be determined in patients without contrast indications. To our knowledge, this is the first study that investigates the potential benefits of contrast-enhanced echocardiography beyond current recommendations for resting echocardiography. Even though the image quality of the included patients would be considered sufficient for image analysis according to today’s guidelines of echocardiography (i.e. excluded if two or more contiguous segments were not visualized on greyscale images), the number of not visualized segments were remarkably reduced from 169 segments to 14 segments when using contrast. However, it should be noted that the most common WMS shift for those NA segments was a change to normal wall motion after contrast analysis (71 % of the segments), implying that improved detection of wall motion abnormalities for contrast-enhanced images is not only restricted to segments with poor image quality, and that decreased regional wall motion indeed can be more evident using contrast even when the endocardial border is considered well visualized. This is not surprising considering the extremely strong enhancement of the endocardial border and detailed visualization of trabecula due to the maximal separation of cavity and myocardium in white and black respectively in the contrast-enhanced images.

There was a significant difference in LV volumes and EF between the different imaging modes, with larger volumes and EF obtained for contrast-enhanced images. It is well known that LV volumes are underestimated when analyzing two-dimensional greyscale images, as the areas between the trabecula within the LV are excluded from the measurements, and that geometrical assumptions are made [11]. Suboptimal image quality can also result in underestimated LV volumes as boundary detection becomes difficult. On the other hand, the difference in measured volumes between greyscale and contrast analysis had no significant impact on patient level, where most patients remained within the same EF classification (≥ 55 %, 45–54 %, 30–44 %, < 30 %). The relatively large EF intervals as well as the fact that both EDV and ESV were underestimated on greyscale images are possible explanations for these results.

The results from the study indicate improved reproducibility for WMSI and EF measurements when using contrast images instead of greyscale images, which is in line with previous findings in patients with mixed image quality [4–6]. In our study, there was a sustained improved reproducibility even in this group with selected image quality. The improved reproducibility was observed both for repeated measurements by experienced readers and by in-experienced readers. Accordingly, contrast administration during echocardiography can therefore be suggested as a complement to minimize the user dependency of image analysis. This suggestion has also been purposed previously [4].

It has been reported in literature that approximately 10 to 15 % of all cardiac patients have indications for contrast-enhanced echocardiography [5, 6]. Following current recommendations, as much as 34 % of the examinations were actually excluded from this study due to being judged to have indication for contrast-enhanced echocardiography. One possible explanation is that this study was performed at a center with high contrast agent usage where the barrier to classify images as suboptimal is potentially lower than for a center with less experience of contrast-enhanced echocardiography, leading to increased exclusion rates.

The retrospective approach of the present study, where an objective gold standard, or alternative imaging modality to confirm the findings of regional wall motion abnormality interpretation is lacking, is a limitation. However, it should be kept in mind the contrast-enhanced imaging is known to improve diagnostic confidence and that several studies have shown that the results from the image analysis based on contrast-enhanced images correlate well with other imaging methods [4, 12–14]. Additionally, a review of the results from previous stress echocardiography and/or coronary angiography examinations have been performed in a subgroup of patients (*n* = 46 patients) where a shift from normal wall motion (WMS = 1 in every segment) to abnormal wall motion was observed when comparing greyscale and contrast analysis. In total, 22 of these patients were confirmed with myocardial ischemia or infarction. When combining the results from the contrast analysis at rest in the present study with previous studies, it was concluded that the wall motion abnormalities overlapped with the diseased areas in 21 patients out of these 22 patients. This confirms that the observed shift in wall motion abnormalities in the present study after contrast analysis may indeed alter the outcome for the patients and improve the diagnostic power of resting echo, even in patients with excellent image quality. Another limitation with the present study is the over representation of patients with ischemic cardiac disease. This is not a true representation of the patient cohort normally referred to echocardiography. In fact, this is a pilot study and we are about to perform a prospective randomized study in order to study the true potential benefit of routine contrast agent use.

This study is a potential first step towards a broader use of contrast-enhanced echocardiography instead of the selective approach used today. From a patient safety perspective it should be kept in mind that contrast-enhanced echocardiography is probably one of the best validated techniques, because of strict protocols and completely blinded readings required for approval of contrast agents [15]. The contrast agents approved for clinical use are well tolerated, and serious adverse reactions are seldom observed [16–18]. Adverse events are in most cases minor (e.g. headache, nausea, altered taste, sensation of heat) and self-resolving. Moreover, the risk for cavitation during ultrasound exposure is limited by applying imaging settings with a mechanical index lower than 0.5. Thus for the individual patient, there is a minor risk to broadening the clinical use of ultrasound contrast agents. The major barrier to overcome is probably more related to the clinical reality, where several obstacles have been pointed out for increased implementation of contrast use, such as absence of experienced specialists, training and accreditation for sonographers to independently perform contrast-enhanced echocardiography [9].

## Conclusions

This study showed that contrast-enhanced echocardiography beyond the current recommendations for contrast agent usage indeed affects assessment of cardiovascular function, in this particular study evidenced by an increased number of detected wall motion and LV structural abnormalities during contrast analysis compared with greyscale analysis. Moreover, contrast-enhancement increased reproducibility for assessment of WMSI and EF measurements in the settings of this study. A prospective study, evaluating the full potential of a more widespread use of contrast agent instead of the current selective approach, is of considerable interest in terms of patient outcomes and increased cost-effectiveness.

## Abbreviations

LV: Left ventricular
WMS: Wall motion score
WMSI: Wall motion score index
EF: Ejection fraction
EACVI: European Association of Cardiovascular imaging
ASE: American Society of Echocardiography
ch: Chamber
NA: Not applicable
EDV: End-diastolic volume
ESV: End-systolic volume
ICC: Intra-class correlation coefficient
